# Arming Mesenchymal Stromal/Stem Cells Against Cancer: Has the Time Come?

**DOI:** 10.3389/fphar.2020.529921

**Published:** 2020-09-29

**Authors:** Giulia Golinelli, Ilenia Mastrolia, Beatrice Aramini, Valentina Masciale, Massimo Pinelli, Lucrezia Pacchioni, Giulia Casari, Massimiliano Dall’Ora, Milena Botelho Pereira Soares, Patrícia Kauanna Fonseca Damasceno, Daniela Nascimento Silva, Massimo Dominici, Giulia Grisendi

**Affiliations:** ^1^ Laboratory of Cellular Therapy, Division of Oncology, Department of Medical and Surgical Sciences for Children & Adults, University-Hospital of Modena and Reggio Emilia, Modena, Italy; ^2^ Division of Thoracic Surgery, Department of Medical and Surgical Sciences for Children & Adults, University-Hospital of Modena and Reggio Emilia, Modena, Italy; ^3^ Division of Plastic Surgery, Department of Medical and Surgical Sciences for Children & Adults, University-Hospital of Modena and Reggio Emilia, Modena, Italy; ^4^ Gonçalo Moniz Institute, Oswaldo Cruz Foundation (FIOCRUZ), Salvador, Brazil; ^5^ Health Institute of Technology, SENAI-CIMATEC, Salvador, Brazil; ^6^ Rigenerand srl, Modena, Italy

**Keywords:** mesenchymal stromal/stem cell, cancer, tumor necrosis factor-related apoptosis-inducing ligand, gene therapy, cell therapy

## Abstract

Since mesenchymal stromal/stem cells (MSCs) were discovered, researchers have been drawn to study their peculiar biological features, including their immune privileged status and their capacity to selectively migrate into inflammatory areas, including tumors. These properties make MSCs promising cellular vehicles for the delivery of therapeutic molecules in the clinical setting. In recent decades, the engineering of MSCs into biological vehicles carrying anticancer compounds has been achieved in different ways, including the loading of MSCs with chemotherapeutics or drug functionalized nanoparticles (NPs), genetic modifications to force the production of anticancer proteins, and the use of oncolytic viruses. Recently, it has been demonstrated that wild-type and engineered MSCs can release extracellular vesicles (EVs) that contain therapeutic agents. Despite the enthusiasm for MSCs as cyto-pharmaceutical agents, many challenges, including controlling the fate of MSCs after administration, must still be considered. Preclinical results demonstrated that MSCs accumulate in lung, liver, and spleen, which could prevent their engraftment into tumor sites. For this reason, physical, physiological, and biological methods have been implemented to increase MSC concentration in the target tumors. Currently, there are more than 900 registered clinical trials using MSCs. Only a small fraction of these are investigating MSC-based therapies for cancer, but the number of these clinical trials is expected to increase as technology and our understanding of MSCs improve. This review will summarize MSC-based antitumor therapies to generate an increasing awareness of their potential and limits to accelerate their clinical translation.

## MSCs and Cancer 

**Figure 1:**
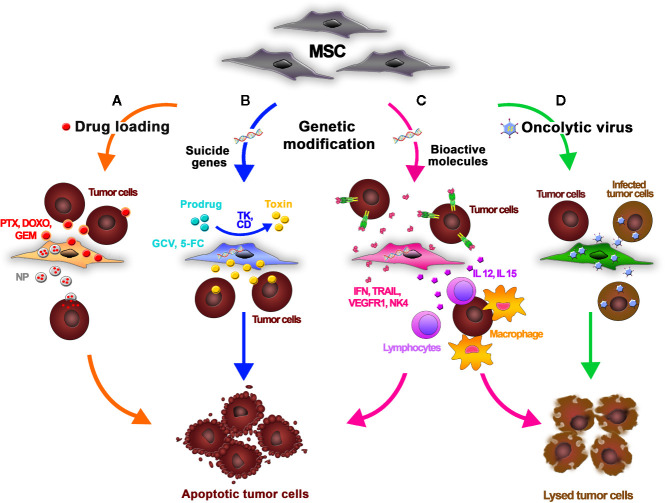
Mesenchymal stromal/stem cells (MSCs) can be functionalized using different strategies to release antitumor agents for cancer treatment. **(A)** An anticancer drug is dissolved in the MSC culture media. MSCs incorporate the chemotherapeutic into the cytoplasm and then release it into the tumor microenvironment. MSCs efficiently absorb doxorubicin (DOXO), paclitaxel (PTX), and gemcitabine (GEM) and release them in their active forms, inhibiting tumor cell growth. MSCs can also take up drug-loaded nanoparticles (NPs), improving their biodistribution. **(B)** Using genetic modification, MSCs can be forced to express suicide genes encoding specific enzymes (e.g., TK, CD) that convert nontoxic prodrugs (GCV, 5-FC) into active derivatives. The prodrugs are systemically administered and then engineered MSCs are intravenously infused. Once injected, MSCs home into the tumor and convert the inactive prodrug into cytotoxic metabolites inside the neoplastic tissue, thus minimizing the off-target toxicity. **(C)** Genetic modification of MSCs can be also performed to induce the production of bioactive molecules and immunomodulatory cytokines such as interferons (e.g., IFN-α, IFN-β, IFN-γ), interleukins (e.g., IL-2, IL-12, IL-15,IL-18), chemokines (e.g., CXC3L1), proapoptotic molecules (e.g., TRAIL), antiangiogenic molecules (e.g., Alpha-1 antitrypsin, NK4, VEGFR1), or molecules with other antitumor properties (e.g., TNF-a, HNF4-a). These proteins can both act directly on tumor cells, inducing apoptosis, and potentiate the host inflammatory response through crosstalk with tumor-infiltrating leukocytes. **(D)** MSCs act as carriers and amplifiers of oncolytic viruses, protecting the viruses from host immune responses and delivering them into tumor sites.

Mesenchymal stromal/stem cells (MSCs) play an important role in restoring tissue homeostasis when injury or damage affects the structural integrity of the tissue (Vizoso et al., 2019). MSCs can be attracted to injury sites by following the gradient of chemo-attractant molecules released by inflammatory cells. At the site of damage, local factors such as hypoxia, cytokines, and Toll-like receptor ligands induce the recruited MSCs to proliferate and express growth factors that accelerate tissue regeneration (Rustad and Gurtner, 2012). Tumors can also mobilize MSCs from distant organs, including bone marrow and adipose tissue, driving their engraftment into the tumor microenvironment by inflammatory signals (Kidd et al., 2012; Chen and Song, 2019). It has been shown that MSCs are strongly recruited by hepatic carcinoma (Xie et al., 2017), breast cancer (Ma et al., 2015), and glioma (Smith et al., 2015). These tumor environments consist of many immune cells, which, alongside cancer cells, secrete soluble factors that can directly regulate MSC chemotaxis and recruitment to damaged tissues. For instance, interleukin (IL)-6 facilitates MSC attraction into tumor sites (Rattigan et al., 2010). An IL-8-dependent recruitment of MSCs was detected in glioma (Ringe et al., 2007), and it has also been shown that platelet-derived growth factor subunit B (PDGFB), vascular endothelial growth factor (VEGF), and transforming growth factor beta-1 (TGF-β1) can induce MSC migration (Schar et al., 2015). Recently, it was revealed that C-X-C motif chemokine receptor 4 (CXCR4) is one of the primary chemokine receptors involved in the enrollment and tumor tropism of MSCs (Kalimuthu et al., 2017). Other chemokines and their receptors with a central role in MSC tumor homing are C-C motif chemokine receptor 1 (CCR1), CCR7, CCR9, C-X3-C motif chemokine ligand 1 (CX3CL1), CXCR5, and CXCR6 (Honczarenko et al., 2006; Feng and Chen, 2009; Bao et al., 2012). In osteosarcoma, it has been shown that stromal cell-derived factor 1 alpha (SDF-1α) is implicated in MSC recruitment to neoplastic tissue. MSCs, in turn, stimulate the migration of osteosarcoma cells by C-C motif chemokine ligand 5 (CCL5)/RANTES secretion (Xu et al., 2009), thereby favoring the spread of cancer by providing metastatic osteosarcoma cells with a favorable microenvironment (Tsukamoto et al., 2012). Due to their well-documented tumor homing, MSCs become part of the tumor stroma, generating fibrovascular cellular elements, including endothelial cells or pericytes, and possibly differentiating into tumor-associated fibroblasts, which are involved in extracellular matrix remodeling (Kidd et al., 2012). The natural and specific ability of MSCs to home and engraft into malignant tissues, along with their immune privileged status, availability, genotypic and phenotypic stability, expandability, and proven safety record in clinical trials, make MSCs the ideal cellular vehicle for the delivery of anticancer agents improving their bioavailability versus more conventional approaches (Housman et al., 2014; Christodoulou et al., 2018). Thus, the engineering of MSCs to induce or enhance the production of biomolecules can counteract cancer growth while (ideally) sparing normal tissues. To achieve this, MSCs can be functionalized to release molecules capable of inducing tumor cell death (**Figure 1**) (Grisendi et al., 2011). The strategies used to convert MSCs into cellular vehicles for anticancer molecules can be classified into two different types. The first category includes nongenetic modifications of MSCs, such as loading with nanoparticle carriers or drugs. The second consists of approaches based on genetic modification of MSCs to induce the expression of anticancer proteins or suicide genes.

## Using Drug-Loaded MSCs to Target Cancer

### Uptake and Release of Chemotherapeutic Agents by MSCs

Because MSCs are relatively resistant to cytostatic and cytotoxic chemotherapeutic agents, they can be loaded with drugs and used for targeted anticancer therapy (**Figure 1A**). One method to do so is to dissolve active compounds in the MSC culture media. The MSCs can incorporate the anticancer drugs into the cytoplasm and release it into the culture medium in a time-dependent manner. Pessina et al. demonstrated that MSCs can efficiently take up the chemotherapeutic agents doxorubicin (DOXO), paclitaxel (PTX), and gemcitabine (GEM) and release them in an active form, resulting in an inhibition of tumor cell growth *in vitro* (Pessina et al., 1999; Pessina et al., 2013; Pascucci et al., 2014; Cocce et al., 2017a; Cocce et al., 2017b). In a leukemia xenograft mouse model, authors demonstrated that PTX-primed MSCs exerted a strong anticancer effect, inhibiting the proliferation of tumor cells and vascularization of the neoplasia (Pessina et al., 2013). The antitumor impact of primed MSCs is currently being investigated in different types of cancer cells. Among others, Bonomi et al. demonstrated in an *in vitro* 3D dynamic culture system that PTX-MSCs suppress the growth of human myeloma cells (Bonomi et al., 2017). Recently, the authors investigated the mechanisms driving PTX release by loaded MSCs, discovering that MSCs can also liberate PTX associated with extracellular vesicles (EVs) acting as “natural anticancer liposomes” (Perteghella et al., 2019). The use of EVs for drug delivery is detailed later in this review.

### MSCs and Nanoparticles

MSCs can also deliver drug-loaded nanoparticles (NPs) to specific target sites (**Figure 1A**). Initial studies introduced MSCs loaded with magnetic and fluorescently labeled NPs in the field of diagnostic. Roger et al. showed that coumarin-6 dye-loaded poly-lactic acid NPs (PLA-NPs) and lipid nanocapsules (LNCs) were efficiently absorbed by MSCs in a concentration- and time-dependent way without influencing the viability and differentiation of MSCs (Roger et al., 2010). These findings prompted the use of NPs loaded with anticancer compounds in MSC-based drug delivery strategies. Originally, NPs were developed to facilitate targeted drug delivery by increasing drug stability; protecting nucleotides from degradation, thus facilitating their entry into the nucleus; and prolonging the effect of the delivered drug, allowing a dose reduction and a possible decrease in side effects. However, their immunogenicity and uneven intratumoral distribution (due to the dense network of collagen and the high interstitial fluid pressure in the tumor environment) often limits their therapeutic potential and clinical application (Li et al., 2016). Nonetheless, the use of MSCs as cellular vehicles for drug-loaded NPs may be an effective option to overcome the limitations in NP biodistribution. MSCs could circumvent the activation of the immune system against NPs, and because MSCs have the ability to migrate within tumor tissue, they could enable entry of NPs into the tumor core (Aggarwal and Pittenger, 2005). Cellular uptake of NPs can be mediated by different mechanisms, including passive transport and active endocytosis (Banerji and Hayes, 2007). NP internalization by MSCs can be facilitated by receptor-mediated uptake and is also affected by the cell proliferation rate, time of exposure, and MSC culture conditions (Sadhukha et al., 2014). To overcome inefficient drug loading by MSCs, NPs can be linked to the cellular membrane of MSCs by covalent conjugation or by physical association obtained by electrostatic and hydrophobic interactions (Li et al., 2011). In addition, smart NPs that control drug cargo release under tumor-specific or external conditions, such as heat, low pH, the presence of enzymes, and light, have also been designed (Lei et al., 2008; Wang et al., 2011; Huang et al., 2013; Qian et al., 2015; Tian et al., 2015). Sadhukha et al. demonstrated an effective tumor-targeting strategy that consisted in engineering MSCs to carry poly(d,l-lactide-co-glycolide) (PLGA) NPs loaded with PTX. In this study, MSCs showed both concentration- and time-dependent absorption of NPs, with scarce impact on key MSC features and a dose-dependent cytotoxicity in lung and ovarian cancer cells both *in vitro* and *in vivo* (Sadhukha et al., 2014). In other studies, PLGA-PTX- or PLGA-DOX-loaded MSCs were found in different cancer types, like prostate, lung and glioma (Pacioni et al., 2015; Levy et al., 2016; Wang et al., 2016). In an orthotopic lung tumor model, Layek et al. demonstrated that MSCs carrying PTX-loaded NPs homed to cancer tissues and created cellular drug storage that released the drug over the time. Although containing significantly lower doses of PTX, treatment with MSCs carrying PTX-NPs resulted in relevant reduction of tumor growth, increased animal survival, and lower toxicity compared to treatment with PTX solution or free PTX-NPs (Layek et al., 2018).

Most of the nanoengineering strategies previously described depend on simple endocytosis of drug-encapsulated NPs into MSCs. The rapid exocytosis of internalized NPs may lead to an adequate drug loading and retention. To increase drug loading in MSCs, Moku et al. developed PLGA NPs conjugated to the cell-penetrating peptide transactivator of transcription (TAT). It was found that TAT functionalization enhanced the intracellular uptake and retainment of NPs in MSCs. Further, treatment with MSCs carrying TAT-functionalized NPs loaded with PTX resulted in a significant inhibition of tumor growth and higher survival in a mouse orthotopic model of lung cancer compared to free drug or NP-encapsulated drug (Moku et al., 2019). In addition to these chemical NP delivery strategies, biological NPs have recently emerged as new MSC-based delivery tools.

## Genetic Modification of MSCs to Target Cancer

Methods to genetically modify MSCs generally use viral vectors, including retroviral, lentiviral, or adeno-associated viral vectors, and DNA plasmids (Marofi et al., 2017). The choice of genetic modification is driven by the aim and the target of the therapy.

### Suicide Genes and MSCs

One approach to cancer treatment involves the delivery of suicide genes by MSCs (**Figure 1B**). After gene manipulation with an appropriate viral vector, MSCs can produce specific enzymes that convert nontoxic prodrugs into active derivatives (Zhang et al., 2015). The prodrugs are administered systemically following intravenous infusion of engineered MSCs. The MSCs home to tumors and convert these prodrugs into cytotoxic metabolites inside the neoplastic tissue, thus minimizing the off-target toxicity. The main advantage of this anticancer approach is the amplification of the toxicity of the drug *via* the bystander effect, which leads to the death of neighboring target cells due to indirect effects caused by engineered MSCs. The cytotoxic effect exerted by the activated prodrug additionally promotes the release of toxic substances that activate immune cells, including cytotoxic T cells and macrophages, leading to more effective cancer death (Zhang et al., 2015). The production of drug metabolites is also highly toxic for the MSC carriers themselves; thus, they die in the process, reducing a remote risk of adverse effects (e.g., transformation events or protumorigenic effects) related to the long-term persistence of homed and nonhomed MSCs in patients at the end of treatment. Drugs with a short half-life or high systemic toxicity, such as ganciclovir (GCV) or 5-fluorouracil (5-FU), may be ideal candidates for gene-directed enzyme prodrug therapy. For these agents, the systemic concentrations required for a therapeutic effect are significantly higher than the tolerated dose. Delivery of the agent directly into the tumor would permit durable effects without the toxicities seen with systemic delivery (Tsao et al., 2004). The most common enzyme-prodrug complexes used in combination with MSCs to target various tumors are herpes simplex virus thymidine kinase complexed with GCV (HSV-TK/GCV system) and yeast cytosine deaminase (CD) with 5-fluorocytosine (5-FC) (Kucerova et al., 2007; Alieva et al., 2012). Adipose tissue-derived MSCs modified to express yeast CD given in combination with 5-FC significantly inhibit the growth of colon cancer in immunocompromised mice (Kucerova et al., 2007). In this approach, MSCs home to the tumor tissue and CD produced by the MSCs converts 5−FC to 5−FU, a tumoricidal chemotherapeutic agent that can then diffuse into the tumor tissue. Co-administration of CD−expressing MSCs and 5−FC was also effective in treating melanoma and human prostate cancer in mouse xenograft models (Kucerova et al., 2008; Cavarretta et al., 2010). Similarly, it has been shown that TRAIL and HSV-TK-modified MSCs in the presence of GCV significantly reduced tumor growth and increased survival in mice bearing highly malignant glioblastoma multiforme (GBM) (Martinez-Quintanilla et al., 2013).

### MSCs Delivering Bioactive Molecules

Genetic modifications of MSCs can be also used to induce the expression of anticancer bioactive molecules (**Figure 1C**). In 2002, MSCs were used for the first time for the targeted delivery of interferon-beta (IFN-β) in an *in vivo* preclinical model of human melanoma (Studeny et al., 2002). MSCs carrying IFN-β were administered into tumor-bearing mice, provoking a significant reduction in tumor growth and an increase in survival compared to the control group. In addition, the authors demonstrated that, after intravenous injection, the engineered MSCs efficiently migrated and engrafted into lung metastases, delivering IFN-β into the tumors. In addition to IFN-β, other therapeutic genes encoding regulatory proteins and immunomodulatory cytokines such as interferons (e.g., IFN-α, IFN-β, IFN-γ), interleukins (e.g., IL-2, IL-12, IL-15, IL-18), and chemokines (e.g., CX3CL1), as well as molecules with proapoptotic functions (e.g., tumor necrosis factor-related apoptosis-inducing ligand [TRAIL]), antiangiogenic activities (e.g., Alpha-1 antitrypsin, NK4, VEGF receptor 1 [VEGFR1]), or other properties (e.g., tumor necrosis factor alpha [TNF-α], hepatocyte nuclear factor 4-alpha [HNF-4α]) have been implemented in preclinical studies (Shah, 2012). There are two advantages of using genes coding for these molecules: first, these proteins may act directly on tumor cells, blocking their proliferation or inducing apoptosis; and second, because of their physiological roles in the immune response, they can potentiate the host inflammatory response *via* crosstalk with leukocytes infiltrating the tumor microenvironment. IL-12 released by engineered MSCs not only exerts a direct antitumor effect in mice with melanoma, lung cancer, and hepatoma, but also activates cytotoxic lymphocytes and natural killer (NK) cells, thereby significantly reducing metastasis (Chen et al., 2008). Similar results were obtained in mouse models of human glioma, renal carcinoma, and Ewing sarcoma (Duan et al., 2009; Gao et al., 2010; Ryu et al., 2011). Umbilical cord MSCs with enhanced IL-15 gene expression significantly suppressed pancreatic tumor growth in mice and stimulated accumulation of NK cells and CD8+ T lymphocytes in the tumor microenvironment, thus supporting the antitumor immune response (Jing et al., 2014). Co-expression of IL-18 and IFN-β by bone marrow MSCs inhibited glioma growth *in vivo* and prolonged the survival of glioma-bearing rats (Xu et al., 2015). One of the most promising antitumor cytokines is TRAIL, which selectively induces apoptosis in cancer cells, but not in most normal cells. TRAIL is the ligand for death receptors that are commonly overexpressed on the membrane of tumor cells. In tumor cells, TRAIL can induce caspase-mediated apoptosis by binding with its receptors death receptor 4 (DR4) and DR5 (Wong et al., 2019). MSCs display resistance to TRAIL due to their low expression of both DR4 and DR5 (Grisendi et al., 2010). In addition, is possible to consistently isolate and modify MSCs from human adipose tissue by minimally invasive surgical procedures (Foppiani et al., 2019; Starnoni et al., 2019). The wild-type gene coding for membrane-bound TRAIL, as well as modified cassettes expressing soluble ligand forms, have been used in MSC-based therapeutic strategies, demonstrating antitumor effects *in vitro* and *in vivo* in a wide variety of human solid neoplasms, including lung cancer, pancreatic cancer, glioblastoma, sarcoma, and hepatocarcinoma (Loebinger et al., 2009; Sasportas LS et al., 2009; Grisendi et al., 2010; Grisendi et al., 2011; Yan et al., 2014; D’Souza et al., 2015; Grisendi et al., 2015; Guiho et al., 2016; Golinelli et al., 2018; Candini et al., 2019; Rossignoli et al., 2019; Spano et al., 2019).

### MSCs and Oncolytic Viruses

In addition to producing therapeutic molecules, MSCs have also been used as carriers and amplifiers for the delivery of oncolytic viruses into tumor sites (**Figure 1D**). An oncolytic virus is an attenuated virus that can infect and kill cancer cells. After infection, cancer cells are destroyed by oncolysis, releasing new infectious virus particles that can stimulate a proinflammatory environment to counteract immune evasion by malignant cells. In this sense, oncolytic viruses not only cause direct destruction of the tumor cells, but also stimulate host antitumor immune responses to help destroy the remaining tumor. Most available oncolytic viruses are engineered to increase tumor tropism and to reduce virulence for nonneoplastic host cells. A number of viruses, including adenovirus, reovirus, measles virus, herpes simplex virus, Newcastle disease virus, and vaccinia virus, have been clinically tested as oncolytic agents (Raja et al., 2018). When oncolytic viruses are systemically administered, the host immune cells recognize viruses as “non-self” and eliminates them before they can reach the tumor site. Autologous MSCs, however, are not recognized as foreign by the host immune system; thus, those incorporating oncolytic viruses can reach the tumor without major limitations (Nakashima et al., 2010). For this reason, introduction of MSCs infected by an oncolytic adenovirus demonstrated better antitumor effects and increased survival compared to direct delivery of the oncolytic adenovirus in xenograft models of ovarian cancer, glioma, and metastatic lung cancer (Yong et al., 2009; Shah, 2012). This effect was due to MSC-mediated defense of the oncolytic virus from host immune system and transport of the viral particles to the tumor location as it has been demonstrated in human glioma, melanoma, breast cancer, lung metastasis, and liver cancer models (Stoff-Khalili et al., 2007; Yong et al., 2009; Xia et al., 2011; Ong et al., 2013). Interestingly, an engineered oncolytic adenovirus carrying a TRAIL gene has been used to treat a mouse model of pancreatic ductal adenocarcinoma (PDAC), a malignant and deadly cancer characterized by an unfavorable prognosis and limited therapeutic options. In this gene therapy strategy, the oncolytic progeny released by engineered MSCs efficiently infects and lyses the tumor cells while simultaneously provoking the apoptosis of noninfected tumor cells *via* the expression of TRAIL molecules. The results collected in this study indicated that in a PDAC mouse model, adipose tissue-derived MSCs delivering TRAIL selectively homed to the tumor site and strongly hampered tumor growth with no evident toxicity or side effects (Kaczorowski et al., 2016).

## MSC-Extracellular Vesicles for Anticancer Drug Delivery

How cancer cells recruit surrounding noncancer cells into the tumor microenvironment remains a relevant and complex topic (Kikuchi et al., 2019). In the last decade, investigators have begun to focus on structures similar to dust particles that are released by cells. These nanoparticles, known as extracellular vesicles (EVs), are now studied worldwide and are recognized to be key carriers of information in cell-to-cell communication. EVs are membrane-bound nanostructures released by cells under physiological and pathological conditions. They are classified based on their size: exosomes (50–100 nm), microvesicles (100–1,000 nm) and apoptotic bodies (over 1000 nm) (Colombo et al., 2014; Lotvall et al., 2014). Present data suggest that tumor cell-derived EVs are biologically important in cancer development, suppressing tumor-directed immune responses and accelerating tumor growth and invasiveness (Kikuchi et al., 2019).

**Figure 2:**
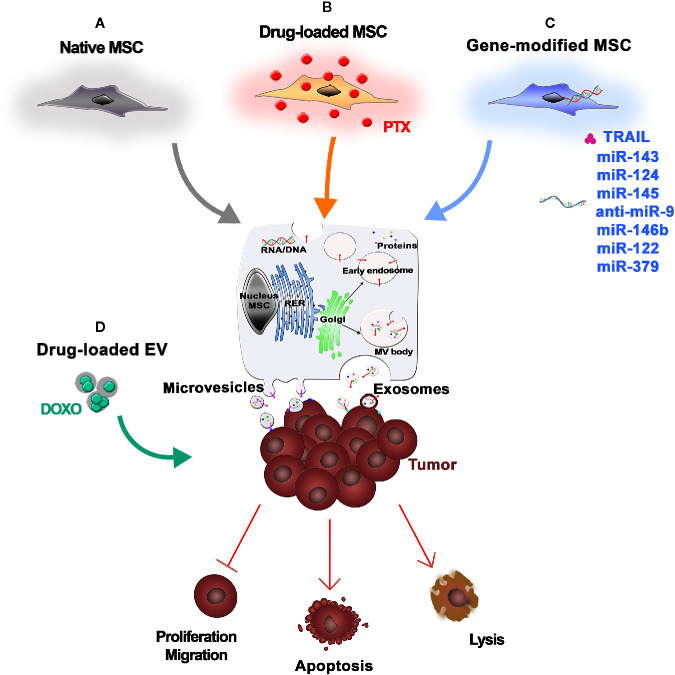
Mesenchymal stromal/stem cell (MSC)-Extracellular Vesicles (EVs) as anticancer drugs. **(A)** Native MSCs are a reliable source of EVs, which are able to influence tumor cell proliferation, migration, and invasion. **(B)** Upon *in vitro* exposure to chemotherapeutic agents [e.g., paclitaxel (PTX)], MSCs internalize and pack the drugs into therapeutic EVs that can efficiently deliver the active drugs to the neoplastic tissue, thus inducing tumor cell apoptosis or lysis. **(C)** MSCs can also be genetically modified to express anticancer molecules (e.g., TRAIL) or miRNAs that can be secreted by MSC-derived EVs. **(D)** Alternatively, EVs are isolated from MSCs and then loaded with drugs or biological cargo by simple diffusion or electroporation.

The previously mentioned synthetic NPs used as drug delivery systems to target cancer (Li et al., 2016) have raised concerns due to their instability after administration, which may be caused by immune reactions, the impact of uncontrolled *in vivo* NP degradation on biocompatibility, and a lack of target specificity (Feliu et al., 2016). In contrast, EVs may be a promising therapeutic tool since they act as intercellular messengers, carrying nucleic acids, lipids, proteins, and miRNA, while maintaining their stability and integrity in circulation, as demonstrated by their presence in most biological fluids (Bruno et al., 2019). EVs are considered nonimmunogenic and are able to protect their cargoes from serum proteases and the immune system, avoiding phagocytosis or degradation (Baek et al., 2019). The specific content of EVs reflects the specific role of the producer cells and determines the biological effect of the vesicles (Isola and Chen, 2017). The current challenge among researchers is to convert this biological message into a therapeutic one. Due to their immunomodulatory capacity, their ability to home to tumor sites, and their robust paracrine factors, MSCs may be a reliable source of EVs for this purpose (**Figure 2**) (Witwer et al., 2019). Growing evidence suggests that MSC-derived exosomes can mediate the transfer of proteins and RNA to tumor cells. However, whether these molecules suppress or promote tumor growth is controversial (Parolini et al., 2009). Interestingly, Roccaro et al. demonstrated that the content and the role of exosomes differ depending on their source. Normal bone marrow MSC (BM-MSC)-derived exosomes are associated with tumor promotion, whereas those derived from multiple myeloma-associated BM-MSCs are linked to tumor suppression (Roccaro et al., 2013). Several studies focused on the intrinsic ability of MSC-derived EVs to counter tumor progression (**Figure 2A**). S. Wu et al. demonstrated the capacity of EVs produced by human Wharton’s Jelly-derived MSCs to abolish tumor cell proliferation *via* G0/G1 phase arrest in a dose-dependent manner (Wu et al., 2013). More recently, an *in vitro* study demonstrated that BM-MSC-derived exosomes can inhibit proliferation, migration, and invasion of pancreatic cancer cells by transporting miR-126-3p, a known tumor suppressor (Wu et al., 2019). Similarly, miRNA-100 seems to be involved in tumor suppression mediated by MSC-derived exosomes. Pakravan et al. demonstrated the ability of MSC-derived exosomes to significantly decrease the expression and secretion of VEGF in a dose-dependent manner in breast cancer-derived cells (Pakravan et al., 2017). However, because MSCs are heterogeneous, MSC-derived EVs may consequently exhibit heterogeneity, which could be an important barrier to their clinical use and should be taken into account (Del Fattore et al., 2015). To circumvent the potential issues caused by the unpredictable effects of native MSC-derived EVs on tumor growth, engineered EVs could be used instead. Current strategies to obtain anticancer EVs are based on the ability of MSCs to take up and release drugs, such as chemotherapeutic agents (**Figure 2B**), or on genetic manipulations of donor cells (**Figure 2C**) (Pessina et al., 2011). Interestingly, Pascucci et al. demonstrated that BM-MSCs exposed to high concentrations of PTX were able to survive and pack PTX into exosomes that could efficiently deliver this active drug to human pancreatic adenocarcinoma cells (Pascucci et al., 2014). The use of exosomes to deliver miRNAs to treat malignant tumors with poor prognosis, such as osteosarcoma or glioblastoma, has also been investigated. *In vitro* studies demonstrated that the introduction of synthetic miR-143 into MSCs increased the secretion of exosome-encapsulated miR-143, which was able to suppress the migration of the osteosarcoma cell line 143B (Shimbo et al., 2014). Further *in vitro* studies investigated the impact of exogenous miRNA mimics delivered by MSCs on glioma cells and glioma stem cells (GSCs) (Bao et al., 2006). MSCs derived from multiple sources can transfer miR-124 and miR-145 mimics to both glioma cells and their GSCs, decreasing migration and self-renewal, respectively (Lee et al., 2013). This evidence demonstrates that exosomes can deliver miRNAs. This ability, combined with their capacity to penetrate the blood–brain barrier, makes exosomes a promising therapeutic tool (Ha et al., 2016). Munoz et al. investigated the role of anti-miR-9-loaded BM-MSC-derived exosomes in reversing the chemoresistance of GBM cells (Munoz et al., 2013). Moreover, *in vivo* studies in a rat brain tumor model demonstrated the efficacy of intratumorally injected miR-146b-expressing MSC-derived exosomes, once again supporting the use of exosomes delivered by MSCs to treat malignant glioma (Katakowski et al., 2013). Likewise, *in vitro* and *in vivo* studies showed that miR-122-transfected adipose tissue-derived MSCs generate exosomes containing miR-122, which is able to increase the sensitivity of hepatocellular tumor cells to chemotherapeutic agents, thereby providing a new therapeutic strategy (Lou et al., 2015). Similarly, the decrease of miR-379 expression in breast cancer is connected to its role as a tumor suppressor. Genetic manipulation of parental MSCs resulted in the release of exosomes containing miR-379 that, upon delivery to the tumor site, showed therapeutic effects (O’Brien et al., 2018). As previously mentioned, TRAIL is a promising anticancer agent (Wong et al., 2019), and TRAIL secretion *via* EVs has been described as a natural approach to deliver messages to near or distant sites that is used by several cell types, including normal T cells upon activation (Monleon et al., 2001) or human placental syncytiotrophoblasts (Stenqvist et al., 2013). Yuan et al. reported an innovative potential anticancer therapy based on EVs expressing surface TRAIL molecules produced by TRAIL-transduced MSCs. These “armed” EVs selectively induced apoptosis in cancer cells, supporting the use of this alternative system for TRAIL delivery (Yuan et al., 2017). The use of MSC-derived EVs in cancer therapy is promising because they, like their producer MSCs, are able to home to cancer sites (Wiklander et al., 2015). However, the exact functions of MSC-derived EVs in tumor biology remain largely elusive, and there are data suggesting that the acidic tumor microenvironment is a key factor that drives the paracrine traffic of EVs within the tumor mass (Parolini et al., 2009). To generate therapeutic EVs, the most common method is to manipulate parental/producer cells to generate EVs containing important cargo, such as regulatory miRNAs or tumor suppressors. However, a passive approach for drug or biological cargo incorporation into EVs is also possible, as EVs can be loaded with drugs by diffusion, or by electroporation when needed (**Figure 2D**) (Raimondo et al., 2019; Vakhshiteh et al., 2019). Although EVs, particularly those derived from MSCs, show promising properties, including high stability, slow clearance, small size, lack of toxicity, and target specificity, many challenges remain to be solved. In particular, exosome isolation would need to be scaled up for clinical applications (Vakhshiteh et al., 2019). This would require a robust standardization of EV manipulation methods and, critically, strict regulations for their clinical use in order to reduce variability in their intracellular content and, consequently, in their biological activities. Large-scale production requires controlled conditions for EV isolation and purification, with attention to donor variability and differences between cell sources. Moreover, the delivery route is critical for EV biodistribution (Raimondo et al., 2019). Several studies support the idea that MSC-derived EVs are able to accumulate in tumors due to their capacity to identify the site of tumors or metastases (Wiklander et al., 2015; Abello et al., 2019). Drug delivery can be further improved by implementing new *ex vivo* modifications, such as surface functionalization. Adding a synthetic multifunctional peptide to EV surfaces substantially increases the ability of the EVs to cross the blood–brain barrier and accumulate in gliomas, enhancing the therapeutic effect of loaded methotrexate (Ye et al., 2018). Despite the advantages of using EVs instead of cells, several challenges remain. For example, potency assays must be developed and appropriate dose findings studies must be conducted (Phinney and Pittenger, 2017). Though the enthusiasm for EVs may be warranted, we are currently far from the safe and controlled clinical use of these biological shuttles.

## Improving MSC Tumor Targeting

**Figure 3:**
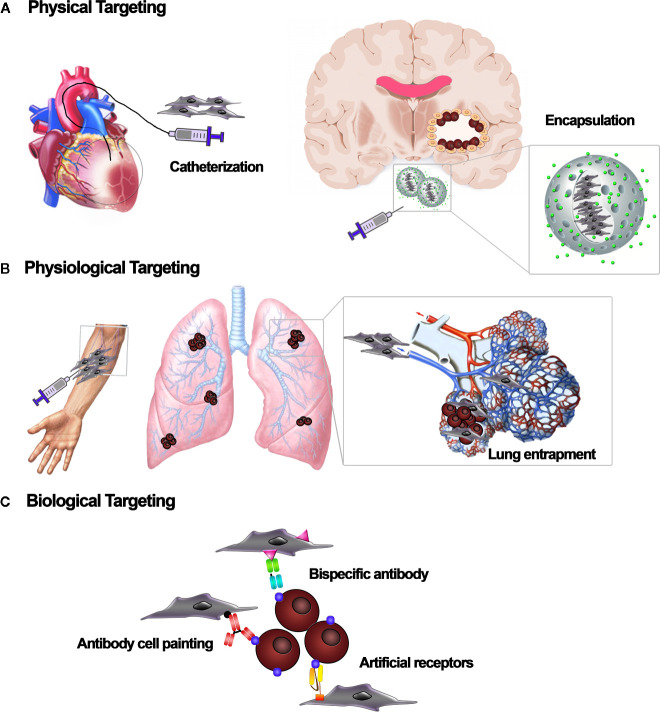
Cell-based targeting strategies. Different targeting strategies to localize mesenchymal stromal/stem cell (MSC) carriers to the tissue of interest. **(A)** Physical targeting relies on the use of devices, such as catheters or scaffolds, to position the cells where they are needed. **(B)** Physiological targeting takes advantage of natural forces that route transplanted cells to specific sites or organs. Based on the infusion site, cells can be physiologically entrapped by the vascular bed of specific tissues. **(C)** Biological targeting strategies embody a range of molecular techniques to target cell vehicles. Adapters such as bispecific antibodies, “cell painting” with antibodies or peptides, and expression of artificial receptors enable the affinity-based retention of cell vehicles at the target site.

MSCs are currently evaluated in clinical trials to treat a variety of diseases, with variable degrees of efficacy. For both locally and systemically injected MSCs, there are issues with MSC fate post-implantation, cell localization, and cell engraftment and survival in the target tissue (Mastrolia et al., 2019). Once locally injected, cells can be lost due to washout, cell death, and rejection by the immune response (Kean et al., 2013). For systemic delivery, the homing ability of MSCs has been showed for several tumors, including gliomas (Nakamizo et al., 2005), breast (Yang et al., 2019), colon (Knoop et al., 2015), ovarian (Komarova et al., 2010), and lung carcinomas (Loebinger et al., 2009). However, only a small amount of systemically administered MSCs effectively reaches the target site (De Becker and Riet, 2016). Current studies indicate that most MSCs accumulate in the lung, liver, and spleen and are subsequently eliminated from the body, which negatively impacts engraftment into the target site (Kean et al., 2013). This suggests that a higher absolute number of cells is needed to guarantee that a sufficient number of MSCs reaches the damage site. However, producing a high number of MSCs is technically challenging in the clinic, in particular for autologous products generated within a cGMP environment. Hence, novel targeting methods are needed to ameliorate MSC engraftment and increase the therapeutic efficacy while reducing the number of cells required and minimizing off-target effects (De Becker and Riet, 2016). MSCs are amenable to various targeting strategies, including physical, physiological, and biological methods aimed at increasing their concentration in the target site (Roth et al., 2008). Physical targeting (**Figure 3A**) involves using either surgical procedures or guiding strategies, such as catheters or external magnets, to place cells directly into the site where the therapy is needed (Arbab et al., 2004; Fiarresga et al., 2015; Silva et al., 2017). Alternatively, therapeutic cells can be restrained in matrices or devices that retain cells at the transplant site (Roth et al., 2008). Notably, Shah et al. reported that MSC encapsulation in a biodegradable, synthetic extracellular matrix significantly increased their retention in the GBM resection cavity while allowing secretion of antitumor proteins (Kauer et al., 2011; Shah, 2013; Duebgen et al., 2014). An additional strategy relies on physiological processes (**Figure 3B**), as the systemic circulation, to move the cells, instead of using active cell-mediated migration (Roth et al., 2008). For example, cells have a tendency to be trapped in the capillary of the lungs. This is a first-pass mechanical barrier to systemic delivery. However, this effect can be exploited to deliver MSC-mediated therapies to the lungs (Hakkarainen et al., 2007; Stoff-Khalili et al., 2007). Recently, biological targeting strategies (**Figure 3C**) have been designed to meet the need for higher target stringency upon systemic infusion of MSCs, especially when the pathology to be treated is widespread, as it is for metastases (Rosenblum et al., 2018). It involves knowledge-driven approaches aimed at improving MSCs homing, binding specificity to target tissue, and retention inside the target environment (Roth et al., 2008). Different strategies have been developed to manipulate MSC homing potential, including modifying the MSC culture conditions to boost the expression of homing-related molecules, engineering the cell membrane to increase homing, and manipulating the target tissue to better recruit MSCs (De Becker and Riet, 2016). For example, the inherent homing potential of MSCs has been exploited by exposing MSCs to glioma-conditioned media (Smith et al., 2015) or to proinflammatory cytokines, such as TNF-α (Egea et al., 2011). The ectopic expression of trafficking machinery components, such as CXCR1, significantly improved MSC tropism toward gliomas secreting high levels of IL-8 (Kim et al., 2011). In addition, radiation augments inflammatory signaling in the cancer site and may be used to improve site-specific MSC migration (Klopp et al., 2007). In parallel to efforts to improve MSC homing, researchers are developing methods to improve MSC affinity for the target site. Affinity-based targeting is dependent on binding interactions and therefore exploits molecules that are exclusively or highly expressed by the cells or tissue that we aim to target and that have affinity for specific receptors on MSCs (Roth et al., 2008). Methods to improve MSC affinity that do not involve genetic modification include antibody- and peptide-based “cell painting” and the use of bispecific antibodies, with applications currently restricted to regenerative medicine (Gundlach et al., 2011; Kean et al., 2013). Most of the work on tumor targeting strategies based on affinity has been done in adoptive immunotherapy, the field in which the highest binding capacity has been achieved, due to immune molecules such as T-cell receptors (TCRs) and their derivatives and chimeric antigen receptors (CARs) (Liu et al., 2019). Affinity-based cell targeting has also recently been applied to MSCs to further optimize their tumor-localizing potential (Golinelli et al., 2018). Balyasnikova et al. genetically modified MSCs to express an artificial receptor (AR) that recognizes EGFRvIII. This allowed the MSCs to specifically target GBM cells expressing EGFRvIII, a mutated form of epidermal growth factor receptor (EGFR) that is not present in healthy tissues but has a high prevalence in GBM. The retention of modified MSCs in EGFRvIII-expressing GBM was significantly increased compared to unmodified MSCs (Balyasnikova et al., 2010). Similarly, Komarova et al. showed that MSC surface modification with an AR that binds to erbB2 increased MSC engraftment and persistence in erbB2-positive ovarian tumors (Komarova et al., 2010). However, evidence supporting targeted anticancer molecule delivery by MSCs expressing an AR remains sparse. The concept of targeted drug delivery as a “magic bullet” was presented in 1908 by Paul Ehrlich and has inspired recent efforts aimed at increasing the concentration of a drug in the tumor site by modulating its affinity for a specific biological target (Strebhardt and Ullrich, 2008). Taking inspiration from strategies used to redirect lymphocyte specificity using CARs or bispecific adaptors, our group coupled affinity and cytotoxicity by genetically modifying therapeutic MSC-TRAIL to express an AR against the disialoganglioside GD2 (Golinelli et al., 2018). The GD2-based targeting allowed MSCs delivering TRAIL to be specifically directed to GD2-expressing cancers, strengthening their adherence to tumor cells. In developing this CAR-based anticancer strategy, we aimed to reach site-specific and lasting retention of MSCs within the tumor bed, thereby effectively delivering proapoptotic TRAIL molecules to GD2-expressing tumors (Golinelli et al., 2018). Combinatorial targeting has recently been applied by Segaliny and colleagues, who produced MSCs that express P-selectin glycoprotein ligand-1 (PSGL-1)/Sialyl-Lewis X (SLEX) together with modified versions of CD and osteoprotegerin (OPG) to treat bone metastases of breast cancer (Segaliny et al., 2019). MSC delivery to bones has been improved through interactions between PSGL-1/SLEX and selectins on activated endothelial cells, megakaryocytes, and platelets in the tumor microenvironment. Once in the tumor niche, engineered MSCs induced local cancer killing through a CD/5-FC suicide gene therapy system and reduced osteolysis by expressing modified OPG (Segaliny et al., 2019). Also noteworthy is the technology developed by Zhu et al. aimed at simultaneously targeting cell proliferation and death pathways in tumor cells using MSCs armed with a bi-functional molecule comprised of a nanobody targeting the EGFR (Enb) and TRAIL (Zhu et al., 2017). EGFR is an excellent target, as it is commonly overexpressed and/or altered in tumor, leading to abnormal cell proliferation and activation of prosurvival pathways. The authors demonstrated that the Enb-TRAIL bi-functional molecule simultaneously engages both EGFR and DR5 on the surface of tumor cells, leading to amplification of the apoptotic signal and proving to be more effective than a combination treatment with Enb and TRAIL. Using an orthotopic resection model of primary glioblastoma, they showed that *in vivo* treatment with encapsulated Enb-TRAIL MSCs reduced tumor growth and considerably increased survival of tumor-bearing mice (Zhu et al., 2017). Although each of the aforementioned tumor targeting approaches individually improves MSC delivery, a combination of different targeting approaches will be likely required to ameliorate both the efficiency and the specificity of cell-based therapies in cancer (Roth et al., 2008).

## MSCs and Cancer Toward the Clinic: Are We There Yet?

Several trials have been designed to study MSCs and their possible implications in cancer treatment. A proportion of these are based on genetically modiﬁed MSCs. However, only four are using MSCs as anticancer vehicles (**Table 1**). Among these trials, the Phase I/II clinical trial TREAT-ME1, with the aim of evaluating the safety and efficacy of MSCs delivering HSV-TK under the control of a CCL5 promoter (Einem et al., 2017). Preclinical studies had demonstrated tumor growth reduction in models of hepatocellular and pancreatic cancer, as well as a reduction in metastases (Niess et al., 2015). Patients enrolled in the study were affected by advanced, recurrent, or metastatic gastrointestinal or hepatopancreatobiliary adenocarcinoma. The clinical trial protocol includes intravenous injection of HSV-TK-engineered MSCs, followed by repeated GCV injections. Intriguingly, this technology is based on CCL5, a chemokine produced by MSCs upon contact with tumor cells, which allows the activation of the CCL5 promoter driving HSV-TK genes only in tumor-infiltrating MSCs, restricting expression of the prodrug-converting enzyme to the tumor microenvironment. This selective activation was introduced to reduce systemic adverse effects. As primary endpoint, they demonstrated acceptable safety and tolerability of the combined cell and gene therapy applied (Einem et al., 2017). An ongoing Phase I clinical trial is studying the best calibrated dose and the side effects of BM-MSCs loaded with the oncolytic adenovirus DNX-2401 in patients affected by recurrent GBM, gliosarcoma, or isocitrate dehydrogenase 1 (IDH1) wild-type anaplastic astrocytoma. DNX-2401 (Delta-24-RGD; tasadenoturev) is a tumor-selective oncolytic adenovirus (clinicaltrials.gov, 2019). The virus has been genetically modified to make it safe for patients and capable of specifically targeting brain cancer cells. This clinical trial has enrolled 36 patients who will be monitored to determine the maximal tolerated dose and local/systemic toxicity (clinicaltrials.gov, 2019). In 2017, a Phase I/II clinical trial (TACTICAL) designed to evaluate the safety and antitumor activity of allogenic MSC-TRAIL in combination with chemotherapy in patients with metastatic nonsmall cell lung cancer (NSCLC) was announced (clinicaltrials.gov, 2017). In Phase I, patients received traditional chemotherapy on the first day, followed by MSC-TRAIL cells on the second day. Each patient received three cycles of treatment at 21-day intervals (clinicaltrials.gov, 2017). Phase I was designed to assess safety and to determine the recommended Phase II dose (RP2D) of MSC-TRAIL when combined with chemotherapy. In Phase II of this trial, which is double-blind, patients will be randomized to the intervention group or the control one. All patients enrolled will be treated by chemotherapy on the first day (clinicaltrials.gov, 2017). However, patients randomized to the intervention group will receive the RP2D of MSC-TRAIL on the second day, while the control group will receive a placebo. The aim of Phase II will be to determine tolerability and efficacy of treatment with MSC-TRAIL in combination with traditional chemotherapy. In summary, TACTICAL will be a key trial to verify the potential of MSC-TRAIL to become a cell-based therapy for patients with advanced lung cancer (clinicaltrials.gov, 2017). A similar therapeutic approach using MSCs to treat PDAC has been announced. In this study, a soluble trimeric and multimeric variant of TRAIL (sTRAIL) is continuously released by adipose (AD)-MSCs and induces apoptosis (Spano et al., 2019). The sTRAIL produced by AD-MSCs that infiltrated the tumor stroma was able to significantly inhibit tumor growth *in vivo*: substantial reductions in tumor mass and in cytokeratin-7-positive cells, as well as an antiangiogenic effect, were observed (Spano et al., 2019). The multiple roles of MSCs in the tumor and their future applications in the clinic, were recently reviewed by Lin and colleagues (Lin et al., 2019), who emphasized the need to focus attention on the molecular mechanism(s) of antitumorigenic activity. Additional studies using MSC-based therapeutic approaches against cancer have been reported. For example, nanodrug carriers can accumulate in tumors due to the leaky tumor vasculature. In 2018, Layek et al. investigated the use of MSCs carrying chemotherapy-loaded NPs as cellular drug carriers. The goal was to generate cellular drug storage capable of migrating to tumors and releasing the drug over a long period of time (Layek et al., 2018). The ability of MSCs to release drugs is commonly employed in cancer therapies. Two registered clinical trials are investigating MSCs for the treatment ovarian cancer. The first one, is a Phase I clinical trial to test the safety and to find the maximum tolerated dose of modified BM-MSCs producing IFN-β that can be given to patients with ovarian cancer (clinicaltrials.gov, 2015). The second, is a Phase I/II clinical trial using AD-MSCs infected with an Edmonston’s strain measles virus genetically engineered to produce sodium iodine symporter (MV-NIS) to treat patients with recurrent ovarian cancer. In Phase I of this trial, the maximum tolerated dose will be defined, and Phase II will consist of intraperitoneal infusion of MV-NIS alone or MV-NIS-modified MSCs. A successful five-year follow-up could lead to an approval for the clinical use of MSCs carrying tumor-killing substances directly to ovarian cancer cells (clinicaltrials.gov, 2014).

**Table 1:** Mesenchymal stromal/stem cell (MSC) clinical trials targeting solid tumors.

Therapeutic Options	Targets	References
1. MSC-HSV-TK	Gastrointestinal cancer	(Niess et al., 2015; Einem et al., 2017)
2. MSC-TRAIL	Nonsmall cell lung cancer (NSCLC)	(clinicaltrials.gov, 2017)
3. MSC-IFN-β	Ovarian cancer	(clinicaltrials.gov, 2015)
4. MSC- MV-NIS	Ovarian cancer	(clinicaltrials.gov, 2014)

In conclusion, the use of MSCs for the treatment of cancer is a promising option. The MSC-mediated delivery of genes, proteins, oncolytic viruses or small molecules in the clinic will take advantage of the abilities of MSCs to be modified and deliver cargoes. While research have to address the MSC tumoral migration/persistence to possibly overcome the limits of nonspecific homing, the potential of combining cells with chemotherapy agents will initiate and write new therapeutic chapters in oncology.

## Author Contributions

GGo, IM, BA, VM, MP, LP, GC, MD’O, MS, PD, and DS participated in the literature search, wrote the manuscript parts, and prepared the figures and tables. MD and GGr conceived the manuscript concept, wrote and final edited the manuscript. All authors contributed to the article and approved the submitted version.

## Funding

This work was supported in part by: Associazione Italiana Ricerca Cancro (AIRC) IG2012 Grant #12755; AIRC IG 2015 Grant 17326 Ministero Italiano Istruzione Università e Ricerca PRIN 2008WECX78, Project “Dipartimenti Eccellenti MIUR 2017” and the Associazione ASEOP.

## Conflict of Interest

MD and GGr hold patents in the field of cell and gene therapy and declare a consultancy role, research funding, and stock ownership with Rigenerand Srl.

The remaining authors declare that the research was conducted in the absence of any commercial or financial relationships that could be construed as a potential conflict of interest
